# Caffeine as a Tool to Explore Active Cognitive Processing Stages in Two-Choice Tasks

**DOI:** 10.1089/caff.2019.0021

**Published:** 2020-06-04

**Authors:** Robert J. Barry, Jack S. Fogarty, Frances M. De Blasio

**Affiliations:** ^1^ Brain & Behaviour Research Institute, University of Wollongong, Wollongong, Australia.; ^2^ School of Psychology, University of Wollongong, Wollongong, Australia.

**Keywords:** caffeine, auditory event-related potentials, Go/NoGo paradigm, active processing

## Abstract

***Background:*** We used caffeine as a tool to explore the active cognitive-processing stages in a simple Go/NoGo task, in terms of the event-related potential (ERP) components elicited by the Go and NoGo stimuli.

***Methods:*** Two hundred and fifty milligrams of caffeine was administered to adult participants (*N* = 24) in a randomized double-blind placebo-controlled repeated-measures crossover study. Two blocks of an equiprobable auditory Go/NoGo task were completed, each with a random mix of 75 tones at 1000 Hz and 75 at 1500 Hz, all 60 dB sound pressure level (SPL).

***Results:*** Major ERP effects of caffeine were apparent in enhancements of the Go N1-1, P3b, and Slow Wave (SW), and the NoGo Processing Negativity, SW, and NoGo Late Positivity.

***Conclusions:*** Novel differential findings indicate the potential of our caffeine as a tool approach to elucidate the functional nature of ERP markers of active cognitive processing in a range of developmental and clinical populations.

## Introduction

The perspective on caffeine as a tool stems from extensive research into the physiological correlates of arousal, the current energetic state of the organism.^1^ Prior research has shown that caffeine reliably increases skin conductance, and globally decreases electroencephalogram (EEG) alpha levels in adults^2–5^ and children,^6,7^ supporting the use of caffeine as a tool to manipulate human arousal levels. Accordingly, the “arousal-as-amplifier” hypothesis would suggest that caffeine will differentially amplify physiological correlates of task-related brain processing, providing valuable insight into common markers of information processing.

The task used in this study was an equiprobable auditory Go/NoGo task in which participants listen to a random series of Go (target; 50% occurrence) and NoGo (nontarget; 50% occurrence) tones, presented at ∼1-second intervals. For task success, each tone must be discriminated, and if a target is identified, participants must execute a button-press response; this is not required for nontargets, so inhibition of that response may be necessary to prevent commission errors. Further processing also occurs as participants evaluate their performance and prepare for the next trial.

**FIG. 1.**
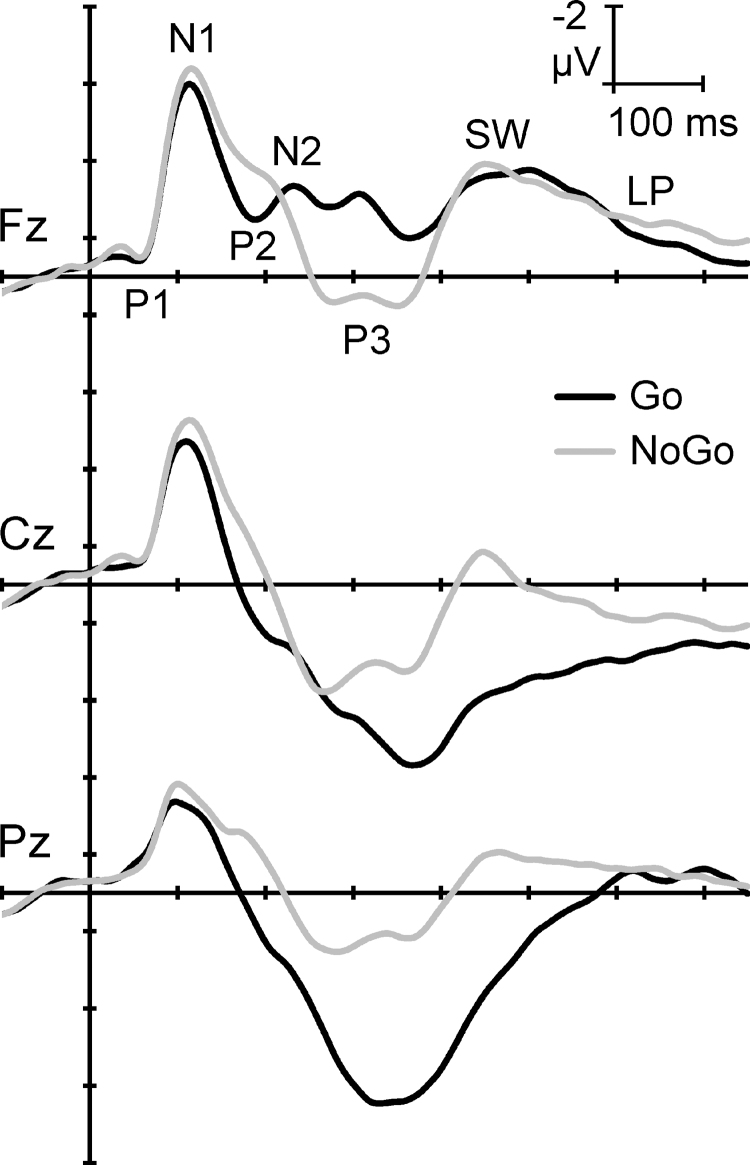
Illustrative adult ERPs in the equiprobable auditory Go/NoGo task, redrawn and relabeled from Fogarty *et al*.^8^ Traditional component peaks are indicated at the frontal midline electrode Fz. Note the similarity in early Go/NoGo waveforms (∼0 to 200 mseconds) and subsequent stimulus-specific component patterning. ERPs, event-related potentials.

These complex brain processes can be reflected in electroencephalographic event-related potentials (ERPs) time locked to Go/NoGo stimulus events. Figure 1 shows the first 750 mseconds of the group-averaged ERPs to Go and NoGo stimuli in this task, redrawn from Fogarty *et al*.^8^ ERPs are similar for the early stimulus processing, but begin to differ when stimulus-specific response requirements are involved. Such waveforms can be decomposed by Principal Components Analysis (PCA, a form of factor analysis), which uses a set of reasonable formal criteria to aggregate time points that vary together into principal components (PCs). It is posited that such PCs provide an approximate model for ERP components, which have been defined as “a temporal pattern of activity in a particular region of the brain that relates in a specific way to how the brain processes information.”^9, p. 141^

**FIG. 2.**
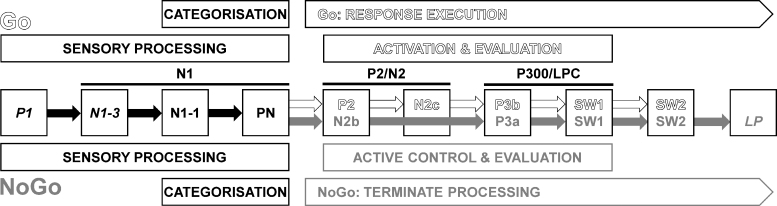
A recent schema of the adult processing involved in the equiprobable auditory Go/NoGo paradigm, redrawn from Fogarty *et al*.^8^ N1-3, N1-1, and PN are subcomponents of N1; N2b and N2c are subcomponents of N2; P3a, P3b, and SW1 are subcomponents of P3 (also called P300 or LPC). Early sensory processing is similar for Go and NoGo; after categorization, further processing of each stimulus type involves different components until later evaluation. Components labeled in *italics* are not always seen. LPC, Late Positive Complex; PN, processing negativity; SW, slow wave.

Figure 2 shows a schema of the processing stages associated with this Go/NoGo task, based on recent outcomes.^8^ In broad terms, stimulus discrimination in each condition is marked by a P1, N1-3, and N1-1 (true N1 components),^10^ and Processing Negativity (PN).^11^ Separate Go (i.e., execution of the Go motor response) and NoGo (i.e., active control and termination of Go response activity) processing then ensue, reflected in the subsequent stimulus-specific components. Go processing is marked by P2, N2c, P3b, and two slow-wave (SW) components (Go SW1 and SW2), whereas NoGo processing is represented by N2b, P3a, NoGo SW1, SW2, and Late Positivity (LP). The conceptualization of this schema has evolved iteratively by linking various behavioral outcomes to the different components.^12–14^ Here instead, caffeine was used as a tool to explore the active processing components.

Presynaptic adenosine receptors are widely distributed over almost all types of neurons,^15^ and the arousal effects of caffeine are mediated by adenosinergic antagonism modulating a variety of neurotransmitter systems.^16–18^ Consequently, ERP component amplitudes have been reported to increase with caffeine, although results are inconsistent.^19–25^ In the Go/NoGo task, Barry *et al*.^19^ found major effects of caffeine on Go P1, P2, and P3b, using traditional (non-PCA) ERP measures. Subsequently, using the standard PCA approach to improve ERP quantification, Barry *et al*.^20^ identified some caffeine enhancement of PN and P3b to Go, but few NoGo effects—only the Go P3b enhancement was consistent with the previous study. Further refinement of the PCA methodology has since been published,^26^ indicating that a single PCA on both Go and NoGo data leads to misallocation of variance between the conditions, effectively smearing condition-specific effects across both. The methodology employed in our previous caffeine PCA study^20^ thus would have resulted in smearing both Go versus NoGo effects, and caffeine versus placebo effects. In this study, we will apply four separate PCAs to avoid such misallocation of variance, expecting to uncover uncontaminated effects that were previously lost.

A general “arousal-as-amplifier” effect of caffeine on ERP components in a particular processing stream would be expected to selectively amplify task-related active component amplitudes—that is, those reflecting brain processes contributing to either Go- or NoGo-specific outcomes. As noted in a recent child study^21^ using the updated PCA methodology, caffeine amplification was apparent in Go PN, N2c, and P3b, and NoGo N1-1 and N2b, and some enhancements were noted in their NoGo-negative SW (NegSW) component (broadly corresponding to the adult SW2) and LP. These effects were used to clarify the sequential processing of children in the Go/NoGo task. However, child ERPs and their underlying cognitive processing differ from those of adults, and these child results may not be generalizable to adults. We now examine such effects of a single oral dose of caffeine in a randomized, double-blind, placebo-controlled repeated-measures crossover design study in adults, using optimized PCA methodology.

## Materials and Methods

### Participants

Twenty-four University of Wollongong students (16 females; 22 right handed) participated voluntarily in exchange for course credit. Their mean age was 21.6 years (standard deviation [SD] = 4.7 years) and they self-identified as nonsmokers and moderate caffeine users (2–4 cups of coffee or equivalent daily). Exclusion criteria included psychiatric illness, a history of seizures and/or severe head injury, and current psychoactive drug use. Participants reported abstaining from caffeine for at least 4 hours before each testing session. Informed consent was obtained following the protocol approved by the local ethics committee.

### Physiological recording

Continuous EEG from 0.15 to 30 Hz was sampled by a 16-bit A/D system (AMLAB II) at 512 Hz from 19 scalp sites according to the “10–20” system (with an electrode cap) and four electro-oculogram (EOG) sites (above/below left eye, beyond the outer canthi of each eye) using tin electrodes. The cap electrode between Fz and Fpz served as the ground, and all data were referenced to linked ears. EEG gain was × 20,000 and EOG gain was × 5000.

### Task and procedure

Participants completed two blocks of an equiprobable auditory Go/NoGo task. Each block contained 150 tones, 75 at 1000 Hz and 75 at 1500 Hz, and all were 50 mseconds in duration (including 5 mseconds rise/fall). Stimuli were presented at 60 dB sound pressure level (SPL) by headphones, in random (within block) order, with a fixed stimulus-onset asynchrony of 1100 mseconds. Using their dominant hand, participants pressed a button in response to the tone designated as the target; the target tone was balanced between subjects, and differed between blocks and sessions within subject. One of two identical gelatin capsules was swallowed with water, containing either 250 mg caffeine or placebo, in a predetermined double-blind randomized order, ∼30 minutes before task commencement to allow plasma levels to maximize.^27^ Approximately 1 week later (mean = 7.5, SD = 1.7 days), participants returned and completed the same procedure with administration of the alternate capsule. The task commenced 35.5 minutes (SD = 6.8 minutes) after placebo ingestion and 36.4 minutes (SD = 6.7 minutes) after caffeine ingestion; these did not differ significantly.

### ERP quantification

The continuous EEG data were EOG corrected^28^ and low-pass filtered (25 Hz, zero-phase shift, 24 dB/octave). Epochs were extracted (−100 to 750 mseconds) relative to Go/NoGo stimulus onset, and baselined across their prestimulus period (−100 to 0 mseconds). Those containing activity exceeding ±75 μV at any scalp site (i.e., muscular or other artifact) and those with incorrect responses (i.e., commission errors in NoGo and omission errors or reaction times outside ±1.5 SD of the individual mean reaction time [RT] in Go), were excluded. Average ERPs were derived (within subjects) at each electrode site for each block (Block 1 and Block 2), capsule (caffeine and placebo), and stimulus (Go, NoGo); this yielded eight (i.e., 2 blocks × 2 capsules × 2 stimuli) 19-channel ERPs for each participant.

Four temporal PCAs (Go caffeine, Go placebo, NoGo caffeine, and NoGo placebo) were carried out in MATLAB^®^ (The Mathworks, v. 8.0.0.783, R2012b) using Kayser and Tenke's^29^ erpPCA functions,^*^ with a heuristic modification from Dien^30^ discussed in Barry *et al*.^26^ The input (ERP) data consisted of 988 cases (26 participants × 2 blocks × 19 channels) and 218 datapoints/variables after the data were half-sampled to 256 Hz; this yielded a case-to-variable ratio of 4.53, approximating Gorsuch's minimum value^31^ (although autocorrelation in EEG data may render this irrelevant). Each PCA used the covariance matrix with Kaiser normalization, and unrestricted Varimax rotation of 218 factors. Placebo PCA components were selected in decreasing order of variance until reaching a floor level of 2%, and identified based on their latency and topographic distribution, their correspondence with the raw ERP, known stimulus-specific properties, and similarity to prior/published data. Caffeine PCA components were similarly extracted to a variance floor level of 2%, but if these did not include a component identified in the corresponding Placebo PCA, that component was included if its variance was above 1%. These are arbitrary limits aimed at minimizing the number of extracted components in an orderly manner.

### Statistical analysis

Paired *t*-tests were used to assess the (one tailed) behavioral effects of caffeine for each assessed error type in Go (omissions, fast, and delayed RTs) and NoGo (commissions), and Go RT measures (mean and variability). Each *t*-test had df = 23.

For each stimulus (Go, NoGo), corresponding components from the placebo and caffeine PCAs were compared using Tucker's^32^ congruence coefficient (*r_c_*); *r_c_* ≥ 0.95 indicates equality and 0.85 ≤ *r_c_* ≤ 0.94 indicates similarity.^33^ Significant grand mean topographic correlations over the scalp sites (*r*[17]) were taken as indicating topographic similarity. Peak Go and NoGo ERP component amplitudes for confirmed corresponding caffeine and placebo components were analyzed from 9 core sites (F3, Fz, F4, C3, Cz, C4, P3, Pz, and P4). However, when analyzing PN, the F3/4, C3/4, and P3/4 electrode pairs were replaced with the corresponding F7/8, T7/8, and P7/8 pairs of the outer electrode rows to account for this component's focus at temporal sites.^34,35^ A two-step analysis was conducted for each assessed component. First, topography was examined in the placebo condition using a two-way repeated-measures multivariate analysis of variance (MANOVA)^36,37^ with topographic factors of sagittal (frontal [F3, Fz, and F4], central [C3, Cz, and C4], parietal [P3, Pz, and P4]), and lateral (left [F3, C3, P3], midline [Fz, Cz, Pz], and right [F4, C4, P4]) dimensions. Planned sagittal contrasts compared frontal (F) and parietal (P) regions, and the central (C) versus frontoparietal (F/P) mean. Lateral contrasts compared left (L) and right (R) hemispheres, and the midline (M) versus hemispheric (L/R) mean. Caffeine effects were then assessed using a three-way repeated-measures MANOVA with factors of drug (caffeine vs. placebo), and the topographic Sagittal and Lateral factors defined above. All contrasts were planned, and did not exceed the degrees of freedom for effect; thus, no Bonferroni-type α level adjustment was required.^38^ All component contrasts had df = (1, 23).

For each component, significant topographic contrasts in placebo were taken as the defining topography. We sought evidence for changes in these defining topographies in the topographic headmap changes from placebo to caffeine. The significance of these was confirmed by statistical interactions of the defining contrasts with Drug; for space reasons, itemization of these are omitted in this study. As we expected that caffeine would increase components actively involved in stimulus processing (i.e., “arousal-as-amplifier” hypothesis), we tested observed increases in global or defining topographic interactions with one-sided probability. Unpredicted decreases in global or defining topographic effects and changes in nondefining topography were tested with two-sided probability.

## Results

### Behavioral data

The behavioral outcomes are presented in Table 1. There were no apparent differences in Fast or Delayed RT errors. Although there were some apparent reductions with caffeine in Go omission errors, NoGo commission errors, mean RT, and RT variability, none of these was significant.

**Table 1.** Behavioral Outcomes, Mean (Standard Deviation)

	Go error %			NoGo error %	Go RT (mseconds)	
Condition	Omissions	Fast RT	Delayed RT	Commissions	Mean	Variability
Placebo	2.1 (2.4)	1.8 (1.8)	7.0 (1.8)	1.4 (1.7)	314.5 (65.1)	61.7 (23.6)
Caffeine	1.4 (2.0)	1.8 (1.8)	6.9 (1.7)	1.1 (1.0)	301.6 (43.8)	58.2 (22.8)
*t*(23)	1.13	0.16	0.21	0.83	1.40	1.32
*p* (one-tailed)	0.136	0.437	0.416	0.208	0.089	0.100

### Event-related potentials

**FIG. 3.**
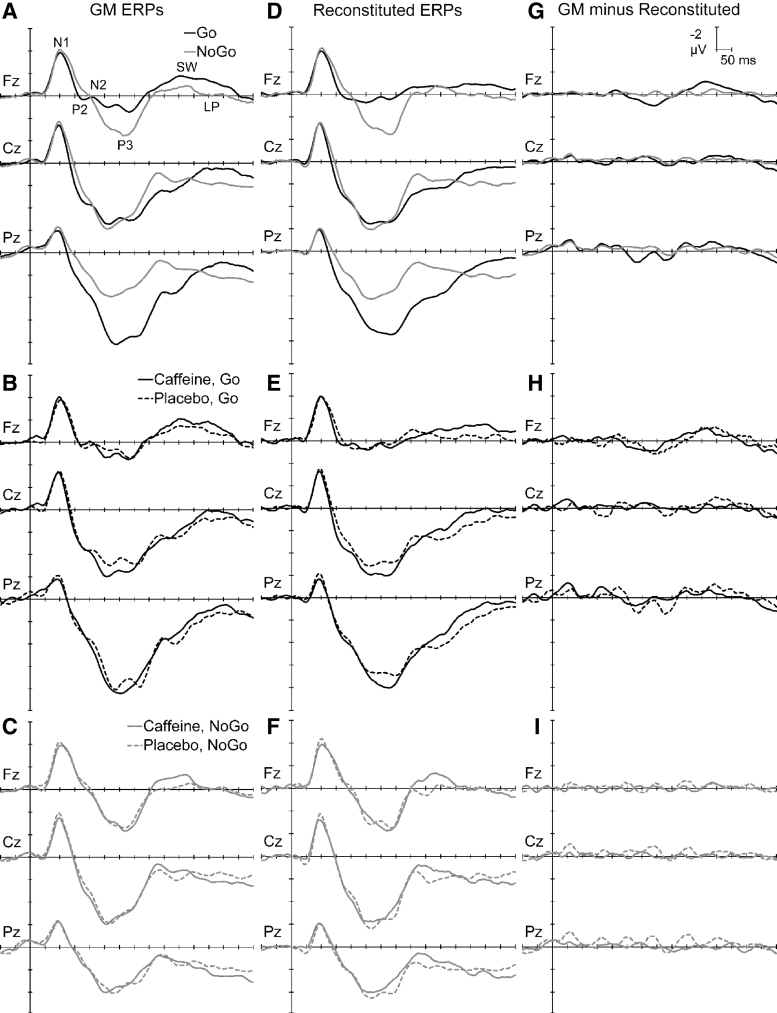
The *left column* shows ERPs at the midline sites for Go vs. NoGo (**A**), and caffeine effects on responses to Go (**B**) and NoGo stimuli (**C**). The *middle column* (**D, E, F**) shows the corresponding reconstituted ERPs from the sum of the PCA-derived components. The *right column* (**G, H, I**) shows the difference between the original and reconstituted ERPs. PCA, principal components analysis.

On average, there were 69.5 (SD = 4.4) accepted trials in each block's mean Go or NoGo ERPs, with no ERP including <57 trials. There were significantly more trials included for NoGo (72.7 ± 2.2) than Go (66.2 ± 3.6) ERPs, *F* = 176.4, *p* < 0.001, η_p_^2^ = 0.88, but these differences are not expected to impact the PCA outcomes. The ERPs for Go and NoGo stimuli at midline frontal (Fz), central (Cz), and parietal (Pz) sites are shown in Figure 3A. There was a frontocentral N1 around 100 mseconds, followed by P3s around 250–400 mseconds with topographic differences apparent between NoGo (frontocentral P3a) and Go (parietal P3b). P2 and N2 were identifiable between N1 and P3 in individual responses, and a frontal-negative/parietal-positive SW and LP followed the P3. Figure 3B suggests that caffeine generally amplified the Go ERP waveforms, while Figure 3C suggests smaller effects on NoGo responses.

### Go PCA outcomes

The first five factors in variance order from the placebo Go PCA (each >2% variance) carried a total of 83.9% dataset variance, while the corresponding components in caffeine (each >1.2% variance) totaled 83.2%. Their sums are displayed in Figure 3E, adjacent to the raw ERPs; summed PCA components display effects similar to those in the raw ERPs (compare right with left). Correlations between the raw ( Fig. 3B) and reconstituted (Fig. 3E) mean Go waveforms ranged between 0.91 and 0.99 for the displayed sites; all were highly significant (*p* < 0.0001), confirming their good approximation. The corresponding difference waveforms for the observed GM minus Reconstituted ERPs are shown in Figure 3H. These suggest that observed discrepancies are random over time and topography, rather than corresponding to any known components.

**FIG. 4.**
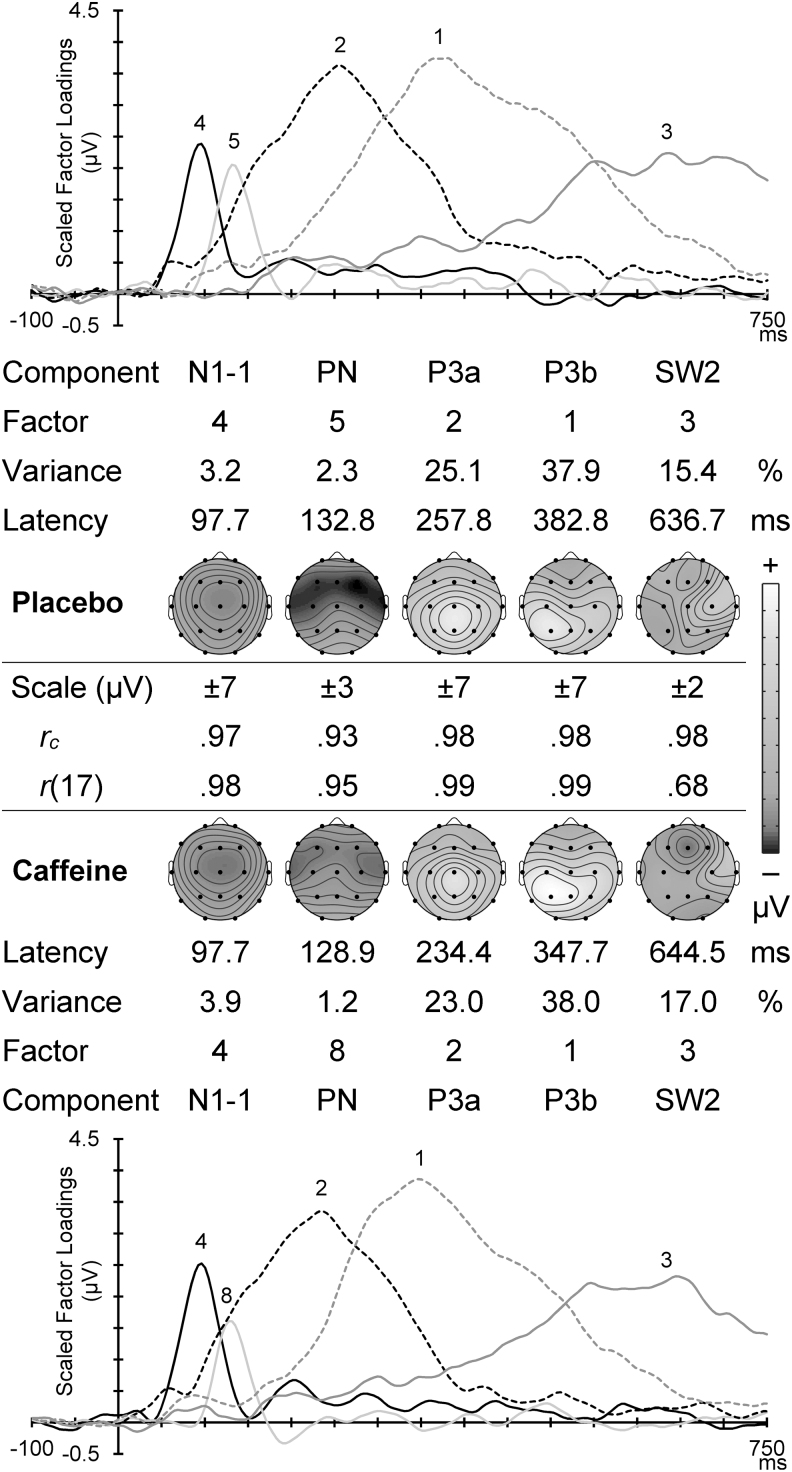
Go PCA scaled factor loadings and component headmaps in placebo (*top*) and caffeine (*bottom*). For each set is shown the factor order, variance carried, and the component peak latency. Between these is shown the scale range for each component headmap, with the congruence coefficient (*r_c_*) and the topographic correlation (*r*[17]) between corresponding components in the two conditions.

Figure 4 displays the scaled factor loadings (showing the timing of activity) and headmaps of peak component amplitudes (showing the spatial topography) for the corresponding Go components in each condition. The first two components are subcomponents of the N1^10^: the frontocentral N1-1 around 100 mseconds, and subsequent temporal PN. These were followed by a midline P3a, a large left parietal P3b, and a late SW (consistent with, and labeled as, SW2.^8^ Between the corresponding caffeine and placebo headmaps is shown the congruence coefficient (all ≥0.93), with the topographic correlation (all ≥0.68; all *p* ≤ 0.001); these confirm their temporal and topographic match between the different drug conditions.

### Go placebo component topographies

Go N1-1 was central (C > F/P: *F* = 22.41, *p* < 0.001, η_p_^2^ = 0.49) with a midline dominance (M > L/R: *F* = 26.99, *p* < 0.001, η_p_^2^ = 0.54). This component was somewhat frontal (F > P: *F* = 3.55, *p* = 0.072, η_p_^2^ = 0.13), and significantly so in the non-midline hemispheric regions (F > P × M < L/R: *F* = 5.02, *p* = 0.035, η_p_^2^ = 0.18). These and other Go component results are illustrated in the placebo headmaps in Figure 4, and their statistics are shown in Table 2. To save space, subsequent topographical results below omit the statistics listed in Table 2.

**Table 2.** Go Component Statistics

Effect	N1-1			PN^a^			P3a			P3b			SW2		
	F	p	η_p_^2^	F	p	η_p_^2^	F	p	η_p_^2^	F	p	η_p_^2^	F	p	η_p_^2^
Placebo topography															
F > P	3.55	0.072	0.13	9.85	**0.005**	0.30	*23.26*	***<0.001***	*0.50*	*46.44*	***<0.001***	*0.67*			
C > F/P	22.41	**<0.001**	0.49	39.78	**<0.001**	0.63	15.29	**0.001**	0.40	6.50	**0.018**	0.22			
L > R										7.82	**0.010**	0.25	*10.12*	***0.004***	*0.31*
M > L/R	26.99	**<0.001**	0.54				22.74	**<0.001**	0.50	*9.01*	***0.006***	*0.28*			
F < P × L > R										32.80	**<0.001**	0.59			
F > P × M < L/R	5.02	**0.035**	0.18	*11.28*	***0.003***	*0.33*	7.86	**0.010**	0.25	31.43	**<0.001**	0.58	15.45	**0.001**	0.40
C > F/P × L < R							3.52	0.073	0.13				6.69	**0.016**	0.23
C > F/P × M < L/R				13.81	**0.001**	0.38	*3.80*	*0.064*	*0.14*	6.37	**0.019**	0.22			
Caffeine effects															
Caff > Plac							*3.90*	*0.060*	*0.14*	9.30	**0.006**	0.29			
Caff > Plac × C > F/P	3.03	**0.095**	0.12												
Caff > Plac × L > R													3.06	0.094	0.12
Caff > Plac × M < L/R										*5.20*	***0.032***	*0.18*	8.19	**0.009**	0.26
Caff > Plac × F > P × L < R							*3.65*	*0.068*	*0.14*				4.94	**0.036**	0.18
Caff > Plac × F > P × M < L/R				9.45	**0.005**	0.29									
Caff > Plac × C > F/P × M > L/R							3.34	**0.080**	0.13						

Bold effects are significant, and italics values indicate a reversal of one relationship indicator in the corresponding effect. Reversals of any two relationship indicators within an effect represent a statistically equivalent effect (e.g., Caff > Plac × M > L/R ≡ Caff < Plac × M < L/R).

^a^ PN component assessed at the temporal (F7, T7, P7, F8, T8, and P8) cf. hemispheric (F3, C3, P3, F4, C4, and P4) sites.

C, central; Caff, caffeine; F, frontal; F/P, frontoparietal; L, left; L/R, hemispheric mean; M, midline; P, parietal; Plac, placebo; PN, processing negativity; R, right; SW, slow wave.

Placebo Go PN was frontocentral, with the frontal enhancement larger in the midline (note that shading in Table 2 indicates a directional reversal of a relative effect listed on the left), and the central enhancement larger in the temporal regions. P3a was midline and centroparietal, particularly in the midline; note that the midline increase of the parietal enhancement reflects the reversal of the two relationships shown on the left of Table 2: F > P × M < L/R ≡ F < P × M > L/R. The central enhancement was also somewhat larger in the right than left hemisphere. P3b was centroparietal and enhanced in the hemispheric regions, particularly the left hemisphere. The central P3b enhancement was larger in the hemispheres, and the parietal enhancement was larger in the midline and left hemisphere. SW2 positivity was largest in the right hemisphere, particularly centrally, and a frontal reduction (negativity) was largest in the midline.

### NoGo PCA outcomes

The first seven factors from the placebo NoGo PCA separately accounted for >2% of the variance, and totaled 83.1% of the dataset variance. Corresponding components in the first seven caffeine NoGo PCA outputs had a total variance of 83.5%. The sums of these in Figure 3F show that the PCA separated components that display sums similar to those in the raw ERPs; correlations between the raw (Fig. 3C) and reconstituted (Fig. 3F) mean NoGo waveforms ranged between 0.98 and 1.00, and were all highly significant (*p* < 0.0001), confirming an excellent approximation. The corresponding difference waveforms for the observed GM minus Reconstituted ERPs are shown in Figure 3I. These suggest that observed discrepancies can be considered solely noise.

**FIG. 5.**
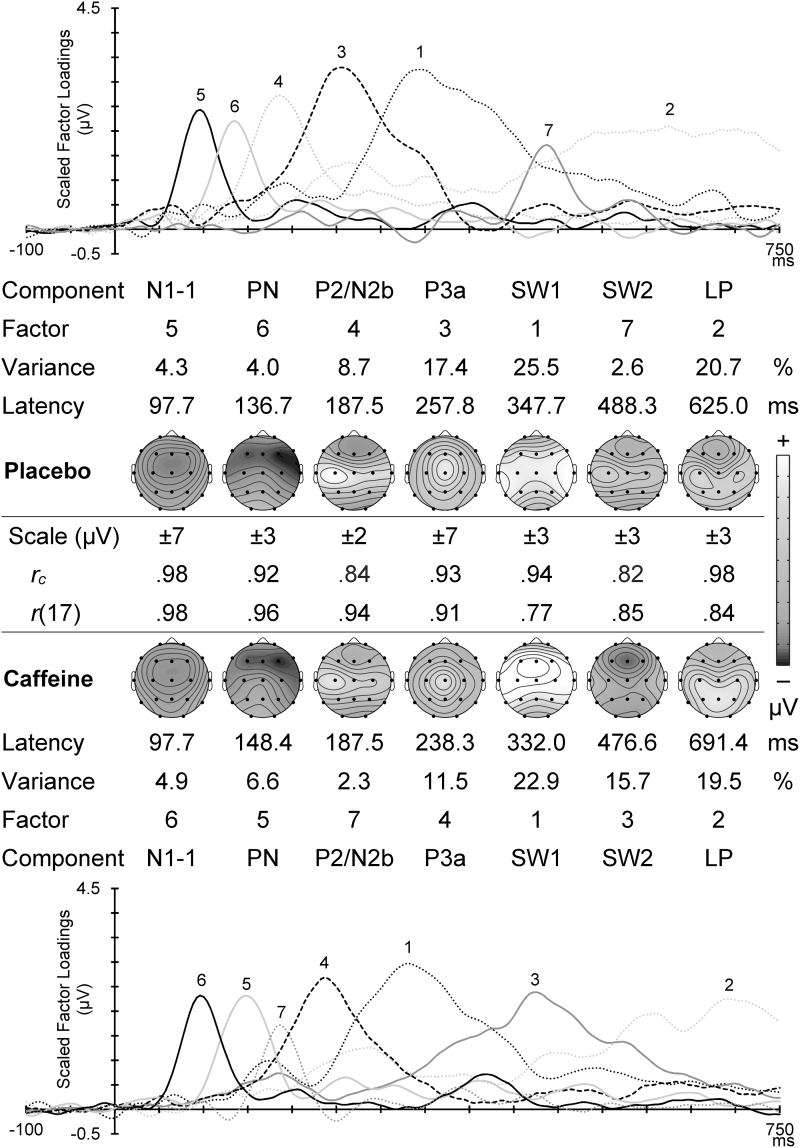
NoGo PCA scaled factor loadings and component headmaps in placebo (*top*) and caffeine (*bottom*). The factor order, variance carried, and the component peak latency are shown for each set. Between these sets is shown the scale range for each headmap, with the congruence coefficient (*r_c_*) and the topographic correlation (*r*[17]) between corresponding components in the two conditions.

Figure 5 displays scaled factor loadings and topographic headmaps of component amplitudes for the matching NoGo components in placebo and caffeine, plotted with the component factor number, percent variance accounted for, and peak latency. A frontocentral N1-1 around 100 mseconds and subsequent PN with temporal focus were followed by a composite P2/N2b, a vertex P3a, a large central SW1, a frontally negative SW (labeled SW2), and LP. Between the corresponding caffeine and placebo headmaps are shown the congruence coefficient (all ≥ 0.82) and the topographic correlation (all ≥ 0.77; all *p* < 0.001); together these confirm the reasonable temporal and/or topographic match of these components.

### NoGo placebo component topographies

NoGo N1-1 was frontocentral with a midline dominance. The frontal enhancement was larger in the right hemisphere, while the central enhancement was larger in the left hemisphere. These and other NoGo component results are reported in Table 3, and evident in the placebo headmaps in Figure 5. NoGo PN was frontocentral, and larger in the right hemisphere, with the frontal enhancement somewhat larger in the midline and the central enhancement larger in the temporal regions. NoGo P2/N2b was more negative frontally (reflecting N2b), particularly in the midline, and more positive centrally (reflecting P2), particularly in the left hemisphere, producing an overall positivity on the left. P3a was central and midline, and these interacted to produce a vertex dominance; there was also some frontal enhancement in the midline. SW1 was central. SW2 was central with some frontal reduction (negativity), somewhat more so in the midline and left hemisphere. LP was centroparietal and hemispheric; the parietal enhancement was larger in the midline, and the central enhancement was larger in the hemispheres, particularly the left hemisphere.

**Table 3.** NoGo Component Statistics

Effect	N1-1			PN^a^			P2/N2b			P3a			SW1			SW2			LP		
	F	p	η_p_^2^	F	p	η_p_^2^	F	p	η_p_^2^	F	p	η_p_^2^	F	p	η_p_^2^	F	p	η_p_^2^	F	p	η_p_^2^
Placebo topography																					
F > P	14.78	**0.001**	0.39	10.48	**0.004**	0.31	*9.49*	***0.005***	*0.29*							*2.99*	*0.097*	*0.12*	*11.92*	***0.002***	*0.34*
C > F/P	17.83	**<0.001**	0.44	59.47	**<0.001**	0.72	57.59	**<0.001**	0.71	38.21	**<0.001**	0.62	4.47	**0.046**	0.16	17.40	**<0.001**	0.43	39.59	**<0.001**	0.63
L < R				7.41	**0.012**	0.24	*11.45*	***0.003***	*0.33*												
M > L/R	10.59	**0.003**	0.32							50.13	**<0.001**	0.69							*11.22*	***0.003***	*0.33*
F > P × L < R	5.93	**0.023**	0.21													3.43	0.077	0.13			
F > P × M > L/R				4.11	0.054	0.15	*9.66*	***0.005***	*0.30*	3.56	0.072	0.13				*3.45*	*0.076*	*0.13*	*19.88*	***<0.001***	*0.46*
C > F/P × L > R	5.08	**0.034**	0.18				4.81	**0.039**	0.17										4.71	**0.041**	0.17
C > F/P × M < L/R				9.53	**0.005**	0.29				*36.89*	***<0.001***	*0.62*							22.56	**<0.001**	0.50
Caffeine effects																					
Caff > Plac										*3.29*	*0.083*	*0.13*	5.02	**0.035**	0.18				3.65	**0.068**	0.14
Caff > Plac × F > P							5.21	**0.032**	0.18	*3.92*	*0.060*	*0.15*	8.01	**0.009**	0.26						
Caff > Plac × C > F/P				*9.15*	***0.006***	*0.28*	*12.09*	***0.002***	*0.34*	12.45	**0.002**	0.35				4.41	**0.047**	0.16			
Caff > Plac × L > R													4.83	**0.038**	0.17						
Caff > Plac × M < L/R													*5.44*	***0.029***	*0.19*	10.95	**0.003**	0.32			
Caff > Plac × F > P × M > L/R				3.95	**0.059**	0.15				*10.94*	***0.003***	*0.32*	19.64	**<0.001**	0.46	*5.80*	***0.024***	*0.20*			
Caff > Plac × C > F/P × L > R										8.40	**0.008**	0.27									
Caff > Plac × C > F/P × M < L/R																9.96	**0.004**	0.30			

Bold effects are significant, and italics values indicate a reversal of one relationship indicator in the corresponding effect. Reversals of any two relationship indicators within an effect represent a statistically equivalent effect (e.g., Caff > Plac × F < P ≡ Caff < Plac × F > P).

^a^ PN component assessed at the temporal (F7, T7, P7, F8, T8, and P8) cf. hemispheric (F3, C3, P3, F4, C4, and P4) sites.

LP, Late Positivity.

### Caffeine effects

**FIG. 6.**
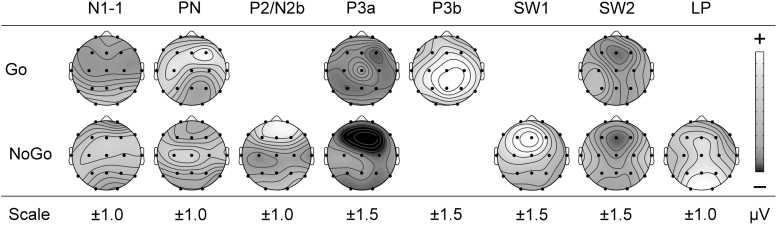
Topographic headmaps for component differences for caffeine minus placebo, with Go outcomes above the NoGo results. *Dark gray* indicates an increase in negativity and *light gray* indicates an increase in positivity. The scale is indicated below each component.

Statistics for the effects of caffeine are shown in the lower parts of Tables 2 (Go) and 3 (NoGo), and illustrated in Figure 6 where the difference headmaps (caffeine relative to placebo) are shown for each component.

For N1-1, caffeine produced a central increase in negativity in Go (one-tailed *p* = 0.048), but had no apparent effect in NoGo. PN showed the opposite caffeine effect: a frontal midline negativity reduction in Go, but a frontal midline negativity enhancement in NoGo (one-tailed *p* = 0.030). NoGo PN also showed a central reduction with caffeine. P2/N2b was apparent only in NoGo, and in this study, caffeine produced reductions in both the N2-related frontal negativity and the P2-related central positivity. For P3a, caffeine produced an increase in the relative central positivity in NoGo (one-tailed *p* = 0.001), and the relative vertex positivity became more apparent in Go (one-tailed *p* = 0.040); however, both of these effects were associated with unpredicted global and focal reductions in positivity outside of these regions. P3b was only apparent in Go, where caffeine was associated with midline (one-tailed *p* = 0.016) and global (one-tailed *p* = 0.003) increases in positivity. The unique NoGo SW1 component also had global (one-tailed *p* = 0.018) positivity caffeine enhancements, as well as focal enhancements in the left hemisphere, and in the frontal, midline, and frontal midline focal regions. The Go SW2 showed caffeine reductions in its right, frontal-right, and midline positivity, while in NoGo, caffeine enhanced both SW2 frontal midline negativity (one-tailed *p* = 0.012) and its central positivity (one-tailed *p* = 0.024; among other things); both Go and NoGo SW2 components thus appear to have had their frontal negativity similarly increased. NoGo LP demonstrated an enhanced global positivity (one-tailed *p* = 0.034).

## Discussion

In our previous work, caffeine was associated with a significant reduction in adult RT and the number of omission errors.^20^ In this study, there were only nonsignificant reductions in RT and Go omission errors. There were also no significant reductions in fast RT errors, delayed RT errors, or RT variability. These could all be Type II errors, but together, the trends observed in this study could support the general impression that caffeine can enhance simple RT performance,^16,39^ although these nonsignificant findings suggest that moderate doses of caffeine do not have substantial activational effects on behavior in the Go/NoGo task.

The ERP components shown in Figure 1 for the adult processing schema were generally obtained in this study, consistent with previous studies in this paradigm.^8,34,40–42^ P1 and N1-3 were not identified, consistent with their italicization in Figure 2. In addition, in the Go processing stream, substantial P2/N2 components were not obtained, and a P3a was unexpectedly found. The paradigm in this study involved each participant completing the task twice, separated by ∼1 week: after placebo and after caffeine, in counterbalanced order. This repetition of the paradigm could partially automate some control processes in the task and underlie the P2/N2 and P3a differences obtained in this study.

The aim in this study was to explore the use of caffeine as a tool to probe the ERP markers of brain processing in the Go/NoGo task. Figure 6 indicates caffeine-produced negativity enhancements in Go N1-1 and NoGo PN. This suggests that stimulus-specific processing has begun at the N1-1 subcomponent stage, and that this selective processing was amplified by caffeine exclusively in the Go condition. Caffeine-enhanced negativity in the NoGo PN also reveals stimulus-specific processing within this common processing stage. Together, these findings support theories linking N1-1 and PN to selective attention and stimulus discrimination^10,43^; speculatively, these effects could also clarify the initial stages of Go and NoGo response selection in adults.^44,45^

A composite NoGo P2/N2b component was identified in this study. Of major interest in this study is the absence of caffeine enhancement of either subcomponent: both the frontal negativity and the central positivity were reduced by caffeine. While the functional significance of P2 is unclear, N2b is frequently linked to cognitive conflict or inhibitory demands.^46,47^ Accordingly, this caffeine-induced reduction in N2b would suggest that adults experience less conflict or inhibitory demand after consuming caffeine. It is plausible that this facilitatory effect is due to a general caffeine-related increase in attentiveness, implied by the N1-1 and PN enhancements.

The identification of a Go P3a in this study conflicts with previous research linking P3a to NoGo response inhibition.^13,14,48,49^ This does not discount its general link to cognitive control, but it does suggest that P3a has a broader function within this paradigm, perhaps associated with attentional control.^50^ Both Go and NoGo P3a components had nonsignificant global reductions in positivity with caffeine, whereas the subsequent Go-specific P3b was globally enhanced by caffeine; see Figure 6. Further research is needed to elucidate the significance of these P3 components. However, these outcomes clearly distinguish P3a and P3b, and reinforce the link between P3b and active target processing in two-choice tasks.^47,50–52^

The centrally positive SW1 was identified only in the NoGo processing stream; Go SW1 was likely not separated from P3b due to their morphological similarity over the scalp electrodes used in this study.^8,53^ SW2 was found in both Go and NoGo processing streams, changing with caffeine from a relatively diffuse central positivity to a frontal-negative, posterior-positive component like the classic SW of Loveless.^54^ Although SW1 and SW2 are relatively novel, following previous SW research,^55,56^ it is tentatively hypothesized that these components represent performance evaluation (SW1) and the subsequent adjustment or preparation needed for the ensuing trial (SW2).^8^ The overall change in both SW1 and SW2 with caffeine appears to have been an increase in frontal activity, suggesting an active processing role, consistent with that hypothesis.

The NoGo LP has been considered to reflect a general increase in cortical positivity, marking a “winding down” of cortical activation following the end of the stimulus-processing chain that is absent in Go because of the extended processing time associated with the behavioral response execution.^12,34^ The caffeine-induced global increase in positivity of the LP found in this study suggests that this disengagement is an active or controlled process (*cf.* passive relaxation).^34^

Apart from Go P3b enhancement, there is little direct comparability of these results with those of our previous adult studies using ERP peak-picking^19^ or PCA component assessment.^20^ The major change with PCA use is the extraction of subcomponents in the dominant N1 and P300/LPC (Late Positive Complex) peaks, but our initial PCA approach^20^ has been shown to be vulnerable to misallocation of variance between the Go and NoGo conditions.^26^ Our improved PCA approach, using separate PCA decomposition of the ERPs from the four datasets (placebo/Go, placebo/NoGo, caffeine/Go, and caffeine/NoGo), reveals the separate impact of these variations in drug (placebo vs. caffeine) and condition (Go vs. NoGo) that have previously been smeared over the combined data. The advantages are apparent in the greater range of effects noted in this study, as summarized in Figure 6.

Several caffeine-related ERP effects identified, in this study, in adults differ notably from the recent findings in children. In children, Barry *et al*.^21^ showed caffeine enhancements in NoGo N1-1 and Go PN, corroborating the stimulus-specific N1 findings in this study; however, in adults, the condition of those effects were reversed (i.e., Go N1-1 and NoGo PN were enhanced in adults). Caffeine also enhanced NoGo N2b in children, but reduced NoGo N2b in adults, suggesting that caffeine does not directly affect the processes underlying N2b. Age-related differences in Go/NoGo task strategy may underpin these findings; children are still developing key executive functions^57,58^ and find it more difficult to control impulses to respond in NoGo trials. Accordingly, the reversed results for the N1-1 and PN enhancements could illustrate that children were focusing (or prioritizing) their attention on identifying NoGo stimuli to reduce commission errors, while adults may have prioritized the identification of Go stimuli. This strategy difference corresponds with the age-specific caffeine effects on N2b, reinforcing studies suggesting that adults do not require substantial amounts of inhibitory control in equiprobable Go/NoGo tasks.^59,60^

## Limitations

Our adult caffeine studies have been carried out in a School of Psychology with a preponderance of female students, resulting in more female volunteers (ranging from 13 to 16 of the 24 participants in each study^19,20^). This varying ratio might also have contributed to variation in the caffeine effects reported. Future studies should seek to obtain an even sex ratio, as in our recent child study.^21^

## Conclusions

Our use of caffeine as a tool to explore sequential processing in adults during the equiprobable Go/NoGo task has produced a number of novel outcomes that clarify ERP markers of the active processing in that task. The caffeine effects on the adult Go/NoGo processing series also differed from those shown in children,^21^ generating a novel suggestion of a developmental shift in strategy; again, illustrating the value of using caffeine as a tool to reveal important cognitive dynamics represented in ERP data. Applying this approach in other paradigms could lead to major advances in our understanding of information processing, and help illuminate disturbances in specific processes associated with a range of developmental/clinical disorders.

## Notes

^*^ http://psychophysiology.cpmc.columbia.edu/software
